# Otalgia revealing McCune-Albright syndrome: A case report

**DOI:** 10.1016/j.amsu.2022.104706

**Published:** 2022-09-20

**Authors:** Achraf Amine Sbai, FatimZahra Es-Salhi, Adil Abdenbi Tsen, Fahd Elayoubi

**Affiliations:** a Department of Ear Nose and Throat, Mohammed VI University Hospital, Medical School, Mohammed the First University, Oujda, Morocco; b Laboratory of epidemiology, Clinical Research and Public Health, Faculty of Medicine and Pharmacy of Oujda, Mohammed the First University, Oujda, Morocco; c Depatement of Cervicofacial Surgery, Mohammed VI University Hospital, Medical School, Mohammed the First University, Oujda, Morocco

**Keywords:** Fibrous dysplasia, McCune-Albright syndrome, otalgia, exophthalmos

## Abstract

**Introduction and importance:**

McCune-Albright syndrome (MAS) is typically described by the asociation of cutaneous (coffee-at-milk spots), endocrine (endocrine hyperfunction most often precocious puberty), and fibrous dysplasia (FD). In 90% of cases, AD manifests itself as a disorder of the craniofacial skeleton, affecting the maxilla, mandible, and even the temporal bone.

**Case presentation:**

We report the case of a 14-year-old girl who presented with complaints of left otalgia with the notion of recurrent otitis evolving for one year, she presents as antecedent an early puberty. At the examination, we found café-au-lait macules, and a slight left exophthalmos without visual acuity decrease. Otoscopic examination showed a narrowing of the external auditory canal (EAC). An audiogram showed conductive hearing loss in the left ear; the air-bone gap was 35 dB. A computed tomography (CT) scan was performed, showing a large "ground glass" appearance of the left temporal region. Given the presence of the cafe-au-lait spot, fibrous dysplasia of the temporal bone, and a history of early puberty, the diagnosis of McCune-Albright syndrome was made.

**clinical Discussion:**

Management of SMA is based on the needs of the individual patient and should be performed by a multidisciplinary team. Management of endocrinopathies is usually medical, with precocious puberty in girls most often treated with aromatase inhibitors. The objectives of the management of craniofacial DF are to correct the functional and aesthetic damage.

**Conclusion:**

McCune-Albright syndrome (MAS) is a rare disease. The involvement of the craniofacial region by FD during SAM is a complicated entity, in its effects and in its management

## Highlights

• McCune-Albright syndrome (MAS) is a rare disease.
• The involvement of the craniofacial region by FD during SAM is a complicated entity, in its effects and in its management.
• the interest of a meticulous clinical examination to guide the diagnosis its recourse to biopsy or genetic study.
• the mechanisms of hearing loss have not been well established, in our paper we will confirm the theory of bone overgrowth around the ossicles and the otic capsule, tested in mice.

## INTRODUCTION

McCune-Albright syndrome (MAS) is a rare disease defined by the presence of at least two of the following features: fibrous dysplasia (FD), typical café-au-lait skin spots and endocrinopathies due to an overproduction of hormones.

Usually the first extra-skeletal manifestation of DSS are café-au-lait spots. Precocious puberty is the most frequent endocrine manifestation, but hyperthyroidism, renal phosphate loss, excess of growth hormone (acromegaly) can also be found [1].

Both bone formation and resorption are affected in FD, this is due to the skeletal stem cell disease. The temporal bone is a common location of FD with variable clinical manifestations including pain, hearing loss, ear infections, ear canal stenosis and cholesteotoma. while the pathophysiology of this broad spectrum remains unresolved [2].

We report the case of a patient who presented with recurrent otitis with chronic otalgia due to a FD, and following this otological findings the diagnosis of MAS was done.

## Observation

A 14-year-old girl presented with complaints of left otalgia with the notion of recurrent otitis evolving since one year, she also presented a slight exophthalmos on the same side without decrease in visual acuity. the patient was born at term with an appropriate height and weight for gestational age (a height of 51 cm and a weight of 3.55 kg), vaccinated according to the national vaccination program, she was no history of trauma, fracture or growth failure, she had attained menarche at 7 years of age. with no family medical history.

On examination, the patient have several café-au-lait macules with irregular and jagged edges on the body, there were no discriminating features of Cushing's syndrome, or acromegaly syndrome, no galactorrhea with a normal thyroid examination, the height at consultation was +0.50 standard deviation (in the normal range) and no extra craniofacial skeletal deformity. Ophtalmic examination found a slight unilateral exophthalmos that was reducible, he visual field test eliminates optic nerve damage.

**Figure 1:**
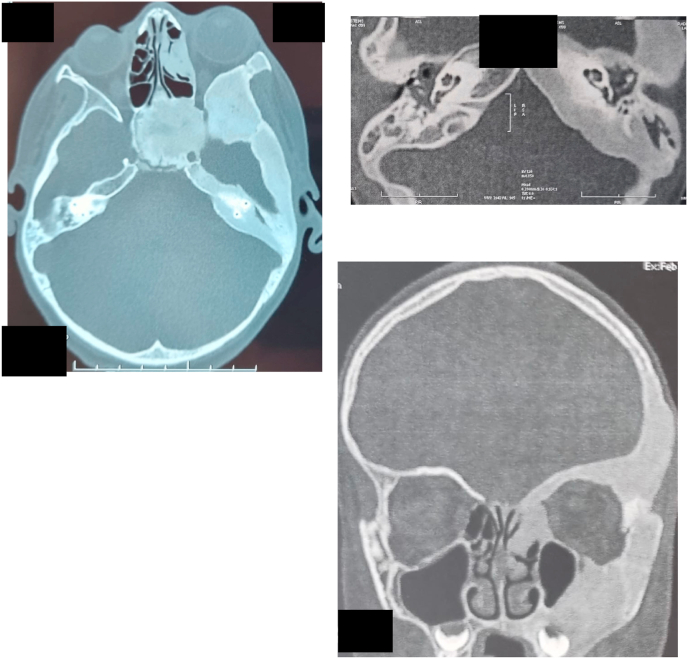
Coronal CT scan shows involvement of the petrous part of the left temporal bone. The superior semicircular canal is surrounded by dysplasia.

The otologic examination foud an inflammation and tenderness of the tragus or pinna, without otorrhea. The otoscope examination showd narrowing of the external auditory canal (EAC) Skin of the external canal was shiny and erythematous, while the skin lining the deep canal shows marked hyperemia covered with a mottled white exudate. An audiogram demonstrated conductive hearing loss in the left ear; the air-bone gap was 35 dB. Computed tom-ography (CT) was performed, showed a large, extensive, homogeneous, "ground glass" appearance of the left temporal area (Figure 1).

Considering presence café au lait spots, fibrous dysplasia of temporal bone and history of precocious puberty, the diagnosis of mccune albright syndrome was established.

Blood biochemistry showed a normal total calcium of 89 (N: 85-105) mg/L, phosphate of 41 (N: 26-48) mg/L, and normal alkaline phosphatase of 90 (N: 42-124)U/L. Thyroid function tests showed a normal thyroid stimulating hormone (TSH) level of 3.2 (normal: 0.3-4.5) IU/mL, and a free T4 of 14 (N: 9 -21) ng/mL. Insulin growth factor 1 and prolactin levels were also normal. Our study has been reported following the SCARE 2020 Checklist criteria [3].

## Discussion

McCune-Albright syndrome (MAS) is typically described by the asociation of cutaneous (coffee-at-milk spots), endocrine (endocrine hyperfunction most often precocious puberty), and fibrous dysplasia (FD) which is a skeletal disorder resulting in deformities, fractures, pain and functional impairment affecting one bone or multiple bones. Patients may develop several combinations of endocrine features (acromegaly, hyperprolactinemia, cushing's syndrome) leading to a complex and highly variable phenotype [4,5].

MAS a rare, non-inherited disorder, results from post-zygotic activating mutations in the guanine nucleotide binding protein alpha (GNAS) gene that encodes the α-subunit of the Gs stimulator protein, resulting in inappropriate cyclic AMP-mediated signaling [4,5].

The MAS mutation affects tissues derived from the endoderm (skin), mesoderm (bone) and ectoderm (endocrine system). This means that the mutation occurs early in embryogenesis, before the separation of the three germ layers, which explains the plymophism of the phenotype of an individual with MAS [4].

The diagnosis of SAM is most often made clinically, by the presence of at least two characteristic elements of the classic triad (cafe au lait spot, precocious puberty and FD), sometimes we find other manifestations of endocrine hyperfunction that can present at different times as acromegaly, hypophosphatemic osteoma, hyperthyroidism and Cushing's syndrome. To make the diagnosis of FD in an isolated lesion of the bone can be done radiologically without recourse to bone biopsy, By the demonstration of a lesion seems "characteristic of FD" which is the basis of the ground glass appearance [4,5].

However, the diagnosis will be problematic in the presence of isolated monostotic FD, in this case we note the need for a biopsy namely the genetic study to identify the somatic mutation of GNAS to confirm the diagnosis [6].

In our case, the diagnosis was made easily given the presence of the classic triad: FD of the temporal bone with typical features (ground glass) the cafe au lait spot at the lumbar level and the history of early puberty.

While the diagnosis of MS should lead to a comprehensive screening evaluation to identify other DF locations and endocrinopathies. In our patient, the clinical, biological and radiological examination did not reveal any other DF localizations or endocrine hypersecretions.

In SAM, FD manifests itself in 90% of cases as a craniofacial skeletal disorder that can lead to facial disfigurement and asymmetry, due to bone expansion, most often involving the maxillary bone and mandible. In some cases, it may involve the temporal bone and cause displacement of surrounding structures such as the orbits, resulting in exophthalmos or visual disturbances, or the external or internal auditory canal and ossicles, resulting in deafness or ear infections. Turbinate FD can cause nasal congestion and obstruction [4].

The present case consulted for an effect of the orbit (exophthalmos) and the auditory canal (narrow canal with external otitis), the craniofacial manifestations were the reason for consultation while knowing that the family ignored the first sign of MAS which is precocious puberty.

The natural history of ear damage and the broad clinical spectrum of DF have not been well established. One study in mice incriminates bony overgrowth around the ossicles and otic capsule as responsible for the severe and progressive hearing loss, but it is not yet known whether this is the same pathophysiology in humans. Another study showed a correlation between hearing loss in MAS patients and growth hormone excess, while another study showed an increased risk of hearing loss in patients with a history of neonatal hypercortisolism. Although our patient did not have acromegaly syndrome or hypercortisolism, this supports the hypothesis that suggests that hearing loss is due to bony overgrowth around the ossicles and otic capsule [2].

Management of SMA is based on the needs of the individual patient and should be performed by a multidisciplinary team. Management of endocrinopathies is usually medical, with precocious puberty in girls most often treated with aromatase inhibitors [7].

The objectives of the management of craniofacial DF are to correct the functional and aesthetic damage. The management depends on the age of the patient, the extent and the clinical behavior of the bone disease [4].

To date, there is no specific treatment for AD. The management of AD is based on pain management. Pain is present in nearly half of patients with craniofacial AD, and treatment relies primarily on bisphosphonates that inhibit osteoclast activity and decrease bone resorption. Although their effect on pain is still controversial, one clinical trial showed no effect of bisphosphonates on pain compared to placebo [1,4].

Close monitoring is a therapeutic option, consisting of a clinical examination (with photographs of the patient) with craniofacial imaging, and in people with signs of damage to the orbit or auditory canal, it must be supplemented by audiological tests and periodic ophthalmological examinations [4,6].

Surgery of Craniofacial FD remains the essential basis of management, it is a conservative surgery with reduction and remodeling procedures, limited by the risk of postoperative regrowth of the bone lesion. It is recommended to screen and treat underlying endocrinopathies prior to surgery [4].

For our patient, we opted for a medical treatment of the otitis externa with clinical monitoring (ophthalmic and otologic examination), audiogram and craniofacial CT in 6 months. The monitoring seems to be sufficient for the moment, during the monitoring if a complication is detected, a decompression surgery will be performed.

## CONCLUSION

SAM is a rare entity whose clinical manifestations are polymorphous. The complication of its management is manifested in the management of FD that fall under several medical and surgical specialties. It is also imperative to screen for and treat DSS-related endocrinopathies, such as growth hormone excess, prior to the management of DF for best results.

## Provenance and peer review

Not commissioned, externally peer-reviewed.

## Availability of data and material

The datasets in this article are available in the repository of the ENT database, Chu Mohamed VI Oujda, upon request, from the corresponding author.

## conflicts of interest

The authors declare no conflicts of interest.

## sources of funding

This research was not funded.

## Ethical Approval

This is a case report that does not require a formal ethical committee approval. Data were anonymously registered in our database. Access to data was approved by the head of the department.

## Consent

Written informed consent was obtained from consent has been obtained from the patient's parents (as legal guardians) for publication of this case report and accompanying images. A copy of the written consent is available for review by the Editor-in-Chief of this journal on request.

## Author contribution

Dr. Achraf Amine SBAI wrote the manuscript.

Dr. FatimZahra ES-SALHI helped in writing.

Pr. Adil Abdenbi Tsen helped in writing and literature review.

Pr. Fahd ELAYOUBI helped in writing, supervised the redaction, revised and approved the final draft for publication.

All authors approved the final version of the manuscript.

## Registration of Research Studies

This is not an interventional study. We only reported the patient’s findings from our database as a case report.

## Guarantor

Dr Achraf SBAI.

## Declaration of/Competing Interest

All authors disclose any conflicts of interest.
